# Hypoglossal nerve stimulation in patients outside the STAR trial criteria: a systematic review

**DOI:** 10.1017/S0022215125000295

**Published:** 2025-07

**Authors:** Pamela Lim Tze Xin, Chuan Hao Gui, Soon Heng Terry Tan

**Affiliations:** 1 Yong Loo Lin School of Medicine, National University of Singapore, Singapore; 2 Department of Otorhinolaryngology, Woodlands Health, Singapore

**Keywords:** Food and Drug Administration, hypoglossal nerve stimulation, stimulation therapy for apnoea reduction trial, upper airway stimulation

## Abstract

**Objectives:**

This study aimed to evaluate if there is a role for hypoglossal nerve stimulation outside the original Stimulation Therapy for Apnea Reduction (STAR) trial criteria.

**Methods:**

This review was conducted using PubMed, Embase and Cochrane Library databases.

**Results:**

Hypoglossal nerve stimulation led to improved outcomes in individuals who fell outside the STAR trial criteria for apnoea-hypopnoea index and body mass index. However, this improvement did not extend to patients with complete concentric palatal collapse or those with a significant central apnoea component.

**Conclusion:**

Hypoglossal nerve stimulation can be effective in patients outside the original STAR trial criteria for certain parameters. Further research is needed to refine patient selection criteria for optimal outcomes.

## Introduction

Obstructive sleep apnoea (OSA) is a condition that affects almost one billion adults worldwide.^1^ It is characterised by recurring episodes of partial or complete airway collapse during sleep. In response, the brain is aroused, the sympathetic system is activated and oxygen is desaturated in the blood.^2^ Individuals with OSA often report snoring, insomnia, lethargy or excessive daytime sleepiness (EDS).^1^^,^^2^

Aside from its repercussions on sleep, it can result in cerebrovascular disorders, cardiovascular disorders, psychological disorders, neurological deficits and decreased work productivity.^3^ Severe OSA is a significant independent predictor of cardiovascular and all-cause mortality.^3^ The effect on EDS has contributed to motor and occupational accidents. Thus, OSA poses a substantial challenge to global health.^3^

The prevalence of OSA has been increasing over time, with a higher prevalence in males compared to females.^2^ This increase can be partly attributed to rising obesity rates, which is a significant risk factor for OSA.^2^ Other risk factors include higher body mass index (BMI), alcohol and exposure to second-hand smoke.^3^

The severity of OSA is usually determined by the Apnoea–Hypopnoea Index (AHI), which is the number of respiratory events divided by the number of hours of sleep on a polysomnography study.^4^^,^^5^ Apnoea is defined as “a drop in peak signal excursion by greater than or equal to 90 per cent of pre-event baseline for greater than or equal to 10 seconds using an oronasal thermal signal (recommended sensor), positive airway pressure (PAP) device flow or an alternative apnoea sensor; without requirement for a desaturation or an arousal”.^5^ Hypopnoea is defined as “a drop in peak signal excursion by greater than or equal to 30 per cent of pre-event baseline for greater than or equal to 10 seconds using nasal pressure (recommended sensor), PAP device flow or an alternative hypopnoea sensor, AND a greater than or equal to 3 per cent oxygen desaturation from the pre-event baseline OR the event is associated with an electroencephalogram (EEG, cortical) arousal.”^5^ Mild, moderate and severe OSA are defined as greater than or equal to 5 to less than 15, greater than or equal to 15 to less than 30 and greater than or equal to 30 (events/hour), respectively.^5^

The gold standard treatment modality is a continuous positive airway pressure (CPAP) machine, in which the user wears a nasal mask overnight during sleep to keep the airway open.^4^ It is indicated in moderate to severe disease independent of symptoms or in lower AHI accompanied with EDS.^4^ Poor tolerance to CPAP has paved the way for the development of alternative treatments.^4^

In terms of other treatment modalities, lifestyle changes and weight loss are recommended for all overweight or obese patients; positional therapy is used in patients whose respiratory events occur nearly exclusively when supine; mandibular advancement devices are indicated in mild to moderate disease, with tongue-base collapse on drug-induced sleep endoscopy (DISE) or CPAP refusal.^4^ Surgical management may be indicated for OSA of any severity, which may include procedures such as uvulopalatopharyngoplasty and maxillomandibular advancement surgery.^6^ Apart from these, hypoglossal nerve stimulation (HGNS) has also emerged as a surgical option.^4^

HGNS, otherwise known as upper airway stimulation, is a device that is implanted in the chest underneath the skin; it initiates electrical impulses which are transmitted to the hypoglossal nerve.^7^ In 1993, Schwartz *et al*. were the first to introduce the concept of HGNS, testing its effects on upper airway collapsibility in cats.^8^ Several companies have produced HGNS systems, including the Apnex device (Apnex Medical, MN USA), the ImThera device (LivaNova, London UK), the Nyxoah Genio device. Thus far, only one company has obtained United States Food and Drug Administration (FDA) approval for their system: the Inspire II (Inspire Medical Systems, MN, USA).^5^

Compared to the aforementioned other modalities, HGNS has a much more stringent criteria for usage. The initial criteria for HGNS was derived from early feasibility studies and the Stimulation Therapy for Apnea Reduction (STAR) trial, which laid the framework for the FDA to determine their candidacy recommendations for Inspire II (Inspire Medical Systems, MN, USA).^5^ In the STAR trial, patients were chosen based on feasibility trials whereby BMI less than or equal to 32 kg/m^2^ and AHI less than or equal to 50 events/h were met with better outcomes, and two small studies (*n* of 7 and 21 patients) which found that HGNS was ineffective if there was palate level complete concentric collapse (CCC) on DISE.^5^

A comparison of the STAR trial criteria and the initial FDA guidelines is presented in Table 1. The STAR trial was a multi-institutional single group trial with 126 patients who were not compliant to CPAP with the following: BMI less than 32 kg/m^2^, AHI more than 20 and less than 50, central or mixed apnoea events less than 25 per cent of all apnoeic events and AHI in non-supine position greater than 10 events/h.^5^ Exclusion criteria included individuals with tonsil size 3 or 4 or palate CCC on DISE.^5^

**Table 1.** Comparison of the STAR trial criteria and initial FDA guidelines

Parameter	Age	AHI	AHI in non-supine position	CPAP	% of central or mixed apnoeas of all apnoeic events	Palate CCC	Tonsil size	BMI
STAR trial criteria ^1^	NS	> 20 and < 50	> 10	Non-compliance	< 25%	None	Excluded if size 3 or 4	< 32
Initial FDA guidelines ^1^	≥ 18	≥ 15	NS	Non-compliance	< 25%	None	NS	NS

AHI = Apnoea-Hypopnoea Index; BMI = body mass index; CPAP = continuous positive airway pressure; CCC = complete concentric collapse; FDA = United States Food and Drug Administration; NS = not specified; STAR = Stimulation Therapy for Apnea Reduction.

Although the STAR trial only included patients with BMI less than 32 kg/m^2^, the FDA indications do not regard BMI as a definitive criterion for candidacy.^5^ The initial FDA criteria suggested that HGNS is indicated for individuals greater than or equal to 18 years old with moderate to severe OSA with failure or intolerance to PAP treatment, less than 25 per cent events that are central or mixed apnoeas and no soft palate CCC.^5^

The eligibility criteria for HGNS are still being evaluated as new literature continues to emerge.^5^ As such, the aim of this study is to evaluate whether there is a role for HGNS in patients who may lie outside the original STAR trial criteria.

## Materials and methods

## Study design and search strategy

This systematic review was conducted in accordance with the latest 2020 Preferred Reporting Items for Systematic Reviews and Meta-Analyses (PRISMA) statement.^9^ To identify relevant studies, a comprehensive search was performed on PubMed, Embase and Cochrane Library databases on 1 July 2024. The search strategy used the following combination of terms “hypoglossal nerve stimulation” or “HGNS” or “HNS” or “upper airway stimulation” or “UAS”, and “Food and Drug Administration” or “FDA” or “Stimulation Therapy for Apnea Reduction” or “Stimulation Therapy for Apnea Reduction Trial” or “STAR” or “STAR Trial”. Only studies published in English were included. Shortlisted studies were reviewed thereafter to assess the suitability for inclusion. To allow for a comprehensive search, we also reviewed the references of all relevant articles.

## Inclusion criteria

Both prospective and retrospective studies were included. Only studies investigating HGNS in patients outside the original STAR trial criteria and those investigating HGNS in a subgroup of such patients were included. Studies were required to report demographic and clinical details, such as patient age, gender, baseline AHI, BMI, upper airway collapse pattern on DISE and surgical technique. For duplicated studies, the most comprehensive and recent report was chosen.

## Exclusion criteria

Our review excluded studies about OSA in the paediatric Down syndrome population and studies exclusively reporting on patients within the STAR trial criteria.

## Results

**Figure 1.**
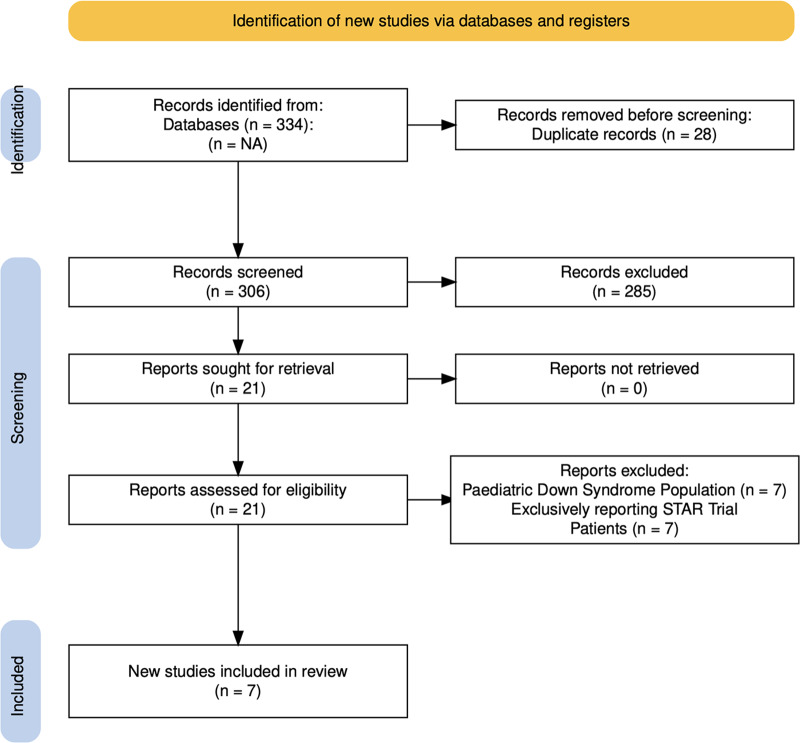
Preferred Reporting Items for Systematic Reviews and Meta-Analyses (PRISMA) 2020 flow diagram.^2^

The initial systematic search identified 334 studies (Figure 1). After removing duplicates, 306 studies remained. Two independent researchers (Lim and Gui) then screened the titles and abstracts of these studies, eventually identifying 21 full-text articles that were relevant to this study. Upon review of the full-texts, seven studies were included in this systematic review.^10^^–^^16^ A flowchart illustrating the study selection process, following the Preferred Reporting Items for Systematic Reviews and Meta-Analyses (PRISMA) guidelines, is presented in Figure 1.

These seven studies consisted of five case series (three prospective, two retrospective), one retrospective case-control and one case report. A total of 88 participants were included in this systematic review. Regarding the level of evidence, one study was classified as level 3, five studies as level 4 and one study as level 5. The characteristics of the included studies are detailed in Table 2

**Table 2.** Characteristics of included studies

Author, year	Study design	Number of patients outside STAR trial criteria	Number of controls	Mean age	Gender	Follow-up after implantation	Device	Inclusion criteria	Exclusion criteria	Measured outcome	Level of evidence
Sarber *et al*., 2020 ^12^	Retrospective case series	18 1. 4 with AHI < 15 2. 4 with AHI > 65 3. 12 with BMI ≥ 32 4. 2 with CAI > 25% of total AHI	NA	63 (range of 44−82) The median age was reported	17 M, 1 F	6 months	Inspire****	AHI ≤ 15 or > 65, BMI ≥ 32, CAI > 25% of total AHI, palatal CCC	-	AHI, ESS, oxyhaemoglobin nadir, arousal index, sleep architecture*****	4
Sarber *et al*., 2019 ^13^	Case report	1 with CAI > 25% of total AHI This patient was already included in the study by Sarber and was not counted again in the total number of patients	NA	Age within range of 60−69 The actual age was not specified	1 M	3 months	Inspire****	-	-	AHI, oAHI, CAI, ESS, O2 nadir	5
Huntley *et al*., 2018 ^14^	Retrospective case-control	40 with BMI > 32	113	59.97 ± 11.50	Not specified	2−2.5 months	Inspire****	Intolerant to CPAP, AHI ≥ 15, UAS implantation, underwent post-operative sleep study	No post-operative sleep study	AHI, ESS, O2 nadir	3
Thaler *et al*., 2016 ^15^	Retrospective case series	9 with AHI > 50 Data was only reported for 7 patients	NA	51.8 ± 13.4	8 M, 1 F	2 months****	Inspire*****	AHI within and above FDA criteria	-	AHI	4
Vanderveken *et al*., 2013 ^16^	Prospective case series	5 with soft palate CCC	NA	55 ± 9	5 M	6 months	Inspire II	Intolerant to CPAP, AHI ≥ 15, BMI < 35	COPD, NYHA class III or IV CHF, neuro-muscular diseases, prior upper airway surgeries unrelated to OSA	AHI, ESS	4
Van de Heyning *et al*., 2012 ^8^^,^^11^	Prospective case series	Part 1: 9 1. 9 with BMI > 32 or AHI > 50 2. 4 with soft palate CCC An overlap was assumed to obtain a total of 9 patients as the study did not specify whether the 9 patients with a BMI > 32 or AHI > 50 overlapped with the 4 patients with CCC.	NA	Part 1: 55.7 ± 8.1*	Part 1: 9 M	6 months	Inspire II	Intolerant to CPAP, BMI < 35, AHI ≥ 25	COPD, NYHA class III or IV CHF, neuro- muscular diseases, prior upper airway surgeries unrelated to OSA	AHI, ODI, ESS, FOSQ, sleep architecture*****	4
		Part 2: 1 with AHI > 50 This patient met*all inclusion criteria except for AHI.		Part 2: 53.6 ± 11.9*	Part 2: 7 M, 1 F**			Intolerant to CPAP, BMI ≤ 32, 20 < AHI < 50, no CCC			
Schwartz *et al*., 2001 ^10^	Prospective case series	6 with AHI > 50	NA	49.9 ± 8.1*	6 M	6 months***	Inspire I	NREM AI > 10, predominantly obstructive apnoeas	Medical, neuro-muscular, otolaryngologic disease	AHI, SaO2, stimulation parameters (amplitude, frequency, pulse width), breathing patterns, sleep architecture*****	4

AHI = Apnoea-Hypopnoea Index (events/hour); AI = Apnoea Index (events/hour); oAHI = Obstructive Apnoea-Hypopnoea Index; BMI = body mass index (kg/m^2^); CAI = Central Apnoea Index; CCC = complete concentric collapse; CHF = congestive heart failure; COPD = chronic obstructive pulmonary disease; CPAP = continuous positive airway pressure; ESS = Epworth Sleepiness Scale; FDA = United States Food and Drug Administration; FOSQ = Functional Outcomes of Sleep Questionnaire; NA = not applicable; NREM = non–rapid eye movement; NYHA = New York Heart Association; ODI = oxygen desaturation index; o2 nadir = oxygen saturation nadir; SaO2 = arterial oxygen saturation; UAS = upper airway stimulation.

* The age of patients outside the FDA criteria was not specified. Thus, the mean age reported encompasses all patients, including those within the FDA criteria.

** The gender of the patient was not specified. Thus, the gender described encompasses all patients, including those within the FDA criteria.

*** A final follow-up appointment was scheduled after this time period, the timing was not specified.

**** Not specified whether Inspire I or II was used.

***** Sleep architecture refers to the following parameters: total sleep time, sleep efficiency, % of time in different stages of sleep.

### Technical specifications of hypoglossal nerve stimulation systems

The following HGNS systems were used: Inspire I stimulating system (Medtronic Inc, Minneapolis, Minn), Inspire II Upper Airway Stimulation (UAS) system (Inspire Medical Systems, Maple Grove, MN). The Inspire I system has an implantable intrathoracic pressure sensor, a programmable pulse generator and a stimulating electrode.^10^ In contrast, the Inspire II system has a respiration sensor, programmable implanted pulse generator (IPG) and a stimulating electrode.^11^ Similarly, both systems use electrodes to deliver an electrical current to the hypoglossal nerve before and during the inspiratory phase, which is detected by their respective sensors. External programming devices are used in both systems to adjust parameters. The key differences are in the electrode design (Inspire I uses a platinum electrode while Inspire II uses a platinum/iridium electrode) and how respiratory signals are detected (Inspire I uses an intrathoracic pressure sensor whereas Inspire II uses the IPG).^10^^,^^11^

### Factors outside the STAR trial criteria

Of the seven studies, three investigated patients with an elevated AHI^10^^,^^11^^,^^15^ and one evaluated patients with both an elevated and reduced AHI.^12^ Three studies assessed patients with elevated BMI.^11^^,^^12^^,^^14^ Two reported on CCC at the soft palate.^11^^,^^16^ Two studies examined outcomes in cases where central apnoea contributed to more than 25 per cent of AHI.^12^^,^^13^

### Outcomes

The majority of the studies did not focus exclusively on patients outside the STAR trial criteria. Consequently, some data could not be extracted as the information was not categorised into subgroups.

The objective outcomes evaluated were AHI, obstructive AHI (oAHI), oxygen desaturation index (ODI), arterial oxygen saturation (SaO2), oxygen nadir (O2 nadir), oxyhaemoglobin nadir, central apnoea index (CAI), arousal index, stimulation parameters, breathing parameters and sleep architecture. It is worthwhile to recognise that AHI measurement serves two purposes: the baseline AHI is a factor that can affect the efficacy of HGNS and the post-operative AHI is measured to assess the effectiveness of HGNS. All of the studies used AHI for the latter purpose. All included studies reported the baseline AHI.

The subjective outcomes assessed included the Epworth Sleepiness Scale (ESS) and Functional Outcomes of Sleep Questionnaire (FOSQ). Additionally, the treatment success rate was also reported, defined as the criteria established by Sher *et al*. (≥ 50 per cent reduction in AHI from baseline and post-treatment AHI < 20).^17^

#### Elevated or reduced AHI as a factor

Of the seven included studies, four^10^^–^^12^^,^^15^ included patients whose baseline AHI fell outside the STAR trial criteria, which is defined as AHI of greater than 20 and less than 50. All four studies^10^^–^^12^^,^^15^ included patients with a pre-operative AHI greater than 50, while only one study^12^ included patients with a pre-operative AHI below 20.

Two of the four studies^10^^,^^15^ concluded that elevated pre-operative AHI levels, even those outside the STAR criteria, are associated with favourable post-operative outcomes. Thaler *et al*.^15^ described how patients with AHI greater than 50 had significant improvements, with mean post-operative AHI of less than or equal to 10. With a baseline mean AHI of 67.2 plus or minus 26.1, AHI was reduced to 5.7 plus or minus 3.9 post-implant, achieving a 91.39 per cent plus or minus 4.46 per cent reduction. Schwartz *et al*.^10^ reported a mean reduction in NREM AHI of 58.1 per cent plus or minus 26.1 per cent following the implantation of HGNS in a subgroup of patients with a baseline AHI of 124.5 plus or minus 25.3. However, data on the total AHI (the sum of NREM AHI and REM AHI) was not reported, and no information regarding treatment success according to Sher’s criteria is available. One study by Sarber *et al*.^12^ reported mixed results, concluding that patients with an AHI greater than or equal to 65 experienced a 50 per cent surgical success rate. A study by Van de Heyning *et al*.^11^ showed contrasting results. In this two-part study design, participants in the first group were initially enrolled using broad selection criteria and evaluated for factors affecting treatment success after HGNS insertion. These factors were then applied in the second group to assess their impact on response. In the first group, patients with an AHI greater than 50 (baseline AHI of 51.1 ± 16.8) experienced poorer outcomes following HGNS insertion compared to those with an AHI between 20 and 50 (baseline AHI of 26.1 ± 5.0).

In the only study evaluating the effect of reduced AHI, Sarber *et al*.^12^ reported a 100 per cent surgical success rate in patients with an AHI less than 15.

#### Elevated BMI as a factor

Two studies^12^^,^^14^ evaluated patients with a BMI greater than or equal to 32, which falls outside of the STAR trial criteria.

Both studies demonstrated that an elevated BMI has positive post-operative outcomes. A case–control study by Huntley *et al*.^14^ reported no difference in post-operative AHI between patients with elevated and non-elevated BMI (6.51 ± 8.26 vs. 5.60 ± 8.95; *p* = 0.441). Success rates were comparable, with 92.30 per cent in the elevated BMI group and 95.40 per cent in the non-elevated BMI group (*p* = 0.345). Additionally, outcomes such as oxygen desaturation nadir and ESS scores did not differ significantly between groups. Sarber *et al*.^12^ presented similar findings, noting a 91.7 per cent surgical success rate among patients with a BMI greater than 32 and a post-operative AHI of 3.4 plus or minus 3.4.

#### Complete concentric collapse as a factor

Two studies^11^^,^^16^ described patients with CCC, which was excluded in the original STAR trial criteria.

Both concluded that CCC is associated with poor post-operative outcomes. Van de Heyning *et al*.^11^ presented the impact of soft palate CCC in four patients, reporting that they were non-responders at six months post-implantation, with AHI increasing from 39.4 plus or minus 14.9 at baseline to 45.2 plus or minus 20.2. In contrast, three patients without CCC responded well, showing a reduction in AHI from 24.9 plus or minus 5.6 to 5.8 plus or minus 4.8. Likewise, Vanderveken *et al*.^16^ found that patients with CCC experienced no significant AHI improvement six months after HGNS, with AHI increasing from 41.5 plus or minus 13.8 to 48.1 plus or minus 18.7 (*p* = 0.44).

#### Central apnoea as a factor

Two studies^12^^,^^13^ reported on patients with elevated central apnoea contributions exceeding the STAR trial criteria (> 25 per cent), where total AHI combines both central and obstructive events, represented by the central apnoea index (CAI) and obstructive AHI (oAHI).^5^

The outcomes were mixed, with unclear effects of HGNS on central apnoea. Although HGNS did not meet the criteria for overall treatment success, it effectively reduced oAHI in both patients, but its impact on CAI varied, decreasing in one patient and increasing in the other. Both patients continued to experience central events post-operatively and developed Cheyne-Stokes breathing.

For the first patient,^12^ AHI decreased from 102.9 (CAI of 35.5, oAHI of 67.4) to 30.8 (CAI of 5.4, oAHI of 25.4) over six months. This patient had both central and obstructive respiratory events at baseline, suggesting a phenotype of OSA with high loop gain and sleep instability. The second patient^13^ initially used CPAP therapy, which was complicated by treatment-emergent central sleep apnoea (TESCA). After subsequently undergoing supraglottoplasty and hyoid suspension, his AHI increased from 44.4 (CAI of 12.5, oAHI of 31.9) to 83.8 (CAI of 78.9, oAHI of 4.9) post-HGNS, while his ESS score improved from 11 to 7, and oxygen saturation nadir rose from 78 to 87.

#### Elevated AHI and elevated BMI as a factor

One study^11^ studied both elevated AHI (> 20) and elevated BMI (≥ 32), which are outside of the STAR trial criteria. It showed that simultaneously elevated AHI and elevated BMI has worse post-operative objective outcomes, but equivocal subjective outcomes.

Van de Heyning *et al*.^11^ conducted a subgroup analysis demonstrating that patients with a baseline AHI less than or equal to 50 and BMI less than or equal to 32 were significantly more likely to achieve successful outcomes (*p* = 0.01), while those not meeting these criteria were less successful. Baseline ESS and FOSQ scores did not differ between groups.

#### Reduced AHI and complete concentric collapse as a factor

One study^16^ evaluated both reduced AHI (< 15) and CCC, which are outside of the STAR trial criteria. A concurrently reduced AHI and CCC was associated with poorer post-operative outcomes.

Vanderveken *et al*.^16^ assessed HGNS outcomes in patients with reduced AHI less than 15, finding a 0 per cent success rate among patients with concurrent palatal CCC, compared to 68.8 per cent among those without CCC. Since AHI less than 15 falls outside the STAR trial criteria, these results suggest that while HGNS may succeed in cases with reduced AHI alone, the addition of CCC significantly reduces success.

## HGNS device malfunction

Only one study^10^ documented instances of device malfunction. These malfunctions were attributed to pulse generator failure, intermittent sensor shutdown, transient asynchronous stimulation due to sensor signal artifact and electrode breakage. It should be noted that this study used the Inspire I device, though it is not specified whether these malfunctions occurred in patients outside the STAR trial criteria.

## HGNS complications

Adverse effects of HGNS were reported in two studies. However, it remains unclear whether these effects occurred in patients outside the STAR trial criteria.

Van De Heyning *et al*.^11^ detailed a case of neck pain and swelling at the incision site post-implantation, which resolved with antibiotics. Another subject required device explantation due to delayed device-related infection. Other minor complications included post-operative pain, stiffness, sore throat, cutaneous stitch abscess, local swelling, fever and lack of tongue response to stimulation within the allowable amplitude range. These all resolved with no intervention. Notably, there was no hypoglossal nerve palsy or pneumothorax.

Sarber *et al*.^12^ described a herpes zoster outbreak on post-operative day 10 and a neck incision skin infection which was treated with oral antibiotics.

## Discussion

The aim of our review was to assess whether HGNS could be beneficial for patients beyond the criteria established in the STAR trial. Published in 2014, the STAR trial cohort showed substantial improvements in objective (AHI, ODI, percentage of sleep spent below 90 per cent saturation) and subjective (daytime sleepiness measured by ESS, snoring levels assessed via bed partner visual analog scores, sleep-related quality of life based on FOSQ) measures of OSA over a five-year period.^16^^,^^18^^,^^19^ At the five-year follow-up mark, 75 per cent of the remaining cohort satisfied Sher’s criteria for treatment success. The success rate was 63 per cent after accounting for those lost to follow-up.^18^

Since then, the landscape of OSA treatment has evolved, with an increasing body of literature supporting the effectiveness of HGNS in broader patient populations. Recent post-approval single-centre and multi-institutional cohort studies have further validated HGNS as a modality which allows for significant improvements in objective and subjective measures. At the three-year mark, the Phase IV German Post-Market Study (GPMS) demonstrated a decrease in median AHI from 28.6 to 10, with 67 per cent of the original cohort reporting an AHI less than 10.^20^^–^^22^ The ADHERE registry, an ongoing prospective observational study, serves as a database of Inspire patients worldwide. It has reported notable improvements in AHI and ESS and higher treatment compliance compared to positive airway pressure therapy. The mean AHI reduced from 35.6 to 10.2 while ESS decreased from 11.9 to 7.5.^23^ At the 12-month mark, 69 per cent met Sher’s criteria.^24^ Along with other studies, the ADHERE registry suggested that HGNS is effective in a larger AHI range (> 15 and < 65), BMI less than 35 and absent palatal CCC on DISE.^5^

This has informed the latest 2023 FDA guidelines, which has expanded the indications for HGNS. The updated criteria now allows for the treatment of individuals greater than or equal to 22 years old with moderate to severe OSA (15 ≤ AHI ≤ 100) who are intolerant to PAP and do not have soft palate CCC. Furthermore, the new guidelines extend eligibility to the following groups provided they meet the above criteria, are not adenotonsillectomy candidates and have been previously considered for other standard alternative treatments. This includes younger patients aged 18 to 21 years old with moderate to severe OSA (15 ≤ AHI ≤ 100) and individuals with Down syndrome aged 13 to 18 years old with severe OSA (10 ≤ AHI ≤ 50). Additionally, this criteria applies to all individuals: central or mixed events must comprise less than 25 per cent of all apnoeic events. The update also specifies a maximum BMI limit of less than or equal to 40.^25^

The success rate of the STAR trial was 63 per cent at the five-year follow-up while the ADHERE registry reported a success rate of 69 per cent at the 12-month follow-up.^18^^,^^24^ In light of the evolving literature and the updated FDA guidelines, this further reinforces the importance of this study, which aims to evaluate whether HGNS can offer benefits to a wider range of patients beyond those initially included in the STAR trial.

Overall, our review found that HGNS led to improved outcomes in individuals who fell outside the STAR trial criteria for AHI and BMI. However, this improvement did not extend to patients with CCC or those with a significant central apnoea component.

### Apnoea-Hypopnoea Index

Determining the likelihood of success with HGNS implantation in patients with an elevated AHI remains challenging. While Schwartz *et al*. and Thaler *et al*.^10^^,^^15^ suggested that HGNS can still be effective in such cases, Sarber *et al*. reported mixed outcomes^12^ and Van de Heyning *et al*.^11^ found it to be ineffective.

It is essential to acknowledge that the study by Schwartz *et al*.^10^ used the NREM AHI as the outcome measure for OSA following implantation. OSA can occur during both rapid eye movement (REM) and non-REM (NREM) sleep, with respiratory events distributed between REM and NREM sleep. Individuals may present with REM-predominant or NREM-predominant OSA.^26^^–^^28^ REM sleep accounts for approximately only 25 per cent of the total sleep duration.^29^ During REM, muscle atonia causes the upper airway to be the most vulnerable to collapse.^26^ It is also characterised by prolonged respiratory events, higher oxygen desaturation and lower respiratory effort compared to NREM sleep.^29^ AHI is calculated as the total number of apnoeas and hypopnoeas per hour during total sleep time. Similarly, the NREM AHI and REM AHI are calculated by the number of events in the respective stages of sleep divided by the duration of NREM and REM time.^30^ In reference to the study by Schwartz *et al*., the total AHI could not be determined because the REM AHI was not reported. Consequently, the NREM AHI alone may not accurately reflect the overall OSA control after implantation as it excludes the REM stage AHI. Moreover, this representation would be further distorted if the patient had REM- or NREM-predominant OSA, which would disproportionately elevate the AHI during REM or NREM sleep, rendering NREM an even less reliable metric.

Among studies evaluating elevated AHI, those with higher baseline AHI values^10^^,^^15^ were associated with greater treatment success when compared to the study by Van de Heyning *et al*.^11^ In the first two studies, Schwartz *et al*.^10^ and Thaler *et al*.^15^ reported baseline AHI values of 124.5 plus or minus 25.3 and 67.2 plus or minus 26.1, respectively. Comparatively, Van de Heyning *et al*.^11^ assessed individuals with baseline AHI values of 51.1 plus or minus 16.8, this lower baseline AHI value could have contributed to the poor outcomes following HGNS in these patients. Similar findings were observed in studies by Kent *et al*. and Renslo *et al*., where a higher AHI baseline was associated with an increased AHI reduction or treatment response.^30^^–^^33^ However, it is important to note that Kent *et al*. reported a mean baseline AHI of 33.8 plus or 15.5, which falls within the STAR trial criteria. Therefore, this finding may not be directly applicable to our study, which involves baseline AHI levels exceeding the STAR trial criteria. In contrast, Renslo *et al*.^31^ did not publish their baseline AHI data, making direct comparison challenging.

Overall, the findings suggest that using HGNS may be beneficial for individuals with elevated AHI, further supporting the FDA’s decision to expand the guidelines to include a broader range of AHI values.

Conversely, one study found that a lower baseline AHI (< 15) was met with successful HGNS implantation.^12^ This suggests that patients with mild OSA can benefit from HGNS. However, the high cost, invasiveness and potential discomfort associated with the procedure may not warrant its use. To prevent overtreatment, it is essential to have a comprehensive discussion with patients about the risks and benefits.^34^

### Body mass index

Two studies^12^^,^^14^ reported successful HGNS implantation in patients with elevated BMI. Contrastingly, the ADHERE registry data has suggested an inverse association between BMI and the effectiveness of HGNS, with a 8.5 per cent decrease in the odds of treatment success for every unit of increase in BMI. However, the cutoff for BMI level has not been well-established.^5^^,^^24^ Kezirian *et al*. showed that patients with a BMI less than 35 experienced a greater reduction in AHI with HGNS. This study employed the use of the Apnex device (Apnex Medical, MN, USA) while our research involved the Inspire device, making direct comparisons less applicable.^35^ Further data is needed to resolve this inconsistency. Nonetheless, a BMI greater than 32 appears to be an indirect predictor of the HGNS response.^36^ BMI has a positive correlation with the probability of palatal CCC.^37^ Thus, if CCC is excluded on DISE, a higher BMI has minimal effect on the success of HGNS.^34^ As such, BMI should be evaluated in tandem with the presence or absence of CCC. Current evidence supports the use of HGNS with BMI less than 40.^5^

Although current FDA guidelines do not include BMI as a definitive candidacy criterion, some insurance policies continue to adhere to the original STAR trial guidelines, which set a BMI threshold of less than 32 for coverage eligibility.^5^ In view of this, cost has emerged as a significant barrier to the widespread adoption of HGNS. The high cost is primarily due to the cost of the device and the cost of the procedure.^38^ The cost of HGNS has been quoted to be approximately 30,000 dollars per individual.^34^ The Inspire system has been demonstrated to be cost-effective, lifetime incremental cost-effectiveness ratio (ICER) of $39,471 per quality-adjusted life year (QALY) for patients meeting the STAR trial inclusion criteria. This is below the commonly accepted cost-effectiveness threshold of $40-50K per QALY. However, it is still significantly less cost-effective than CPAP, which has an ICER of $15,915 per QALY.^38^ More research should be done to determine the cost-effectiveness of HGNS in patients outside the STAR trial criteria. This would help to inform public health policies and insurance coverage, potentially enabling more individuals to access this treatment modality, particularly those with a high BMI who are currently excluded from coverage. In cases where CCC is absent, these individuals may still benefit from the treatment, as it could prove effective despite their BMI.

### Complete concentric collapse

Our findings suggest that HGNS is ineffective for patients with CCC and may even exacerbate OSA.^11^^,^^16^ On DISE, CCC is the strongest contraindication to HGNS. Therefore, even when other criteria for HGNS are satisfied, an anatomical pattern of CCC is a strong indicator of potential treatment failure.^34^ This pattern of collapse is widespread, affecting 20 to 25 per cent of patients who cannot tolerate CPAP and may be candidates for HGNS.^34^^,^^39^ There are currently no multi-institutional studies that have showed HGNS success in patients with CCC.^5^ Of note, the absence of CCC has been a common requirement in the STAR trial criteria, the original FDA guidelines and the latest 2023 FDA guidelines. In conjunction with our findings, we conclude that CCC is a significant factor that renders HGNS ineffective.

### Central apnoea

The requirement that central or mixed events comprise less than 25 per cent of all apnoeic events has been consistently applied in the STAR trial criteria, the original FDA guidelines and the latest 2023 FDA guidelines. The two studies by Sarber *et al*.^12^^,^^13^ highlighted instances where HGNS failed in patients with a significant central apnoea contribution to their total AHI. The effects of HGNS on these patients varies. In one case, the CAI increased, while in the other, it decreased. Furthermore, one patient developed central sleep apnoea after implantation, which was hypothesised to be due to TECSA. However, both patients showed a reduction in oAHI after implantation.^12^^,^^13^ Wang *et al*. hypothesised that OSA patients with severe daytime sleepiness might be more susceptible to developing TESCA, with an ESS score of 16 or more associated with severe sleepiness.^40^^,^^41^ In contrast, the patient who developed TESCA in the study by Sarber *et al*. had an ESS of 11. The ESS score of the other patient who did not develop TESCA was not reported.^13^ This suggests that severe daytime sleepiness may not fully explain the risk of developing TESCA. Further research is required to understand the mechanisms behind these variable outcomes.

### Use of HGNS in the paediatric Down syndrome population

Our study excluded articles on HGNS in paediatric Down syndrome patients due to the lower prevalence of OSA in children compared to adults, which is estimated to be 1–3 per cent.^5^ Although recent FDA guidelines has extended the use of HGNS to individuals with Down syndrome aged 13 to 18 years old with severe OSA (10 ≤ AHI ≤ 50), this is a relatively new and specific subgroup. By focusing our study on adult populations, in which OSA is more prevalent, we endeavoured to generate findings that are more broadly applicable to the larger OSA adult population. Further studies on paediatric Down syndrome patients are warranted but were beyond the scope of our current investigation.

### Limitations

In terms of limitations, only one study included was classified as level 3 evidence, which compared two study arms. The remaining studies were predominantly level 4, with one being level 5. This reflects the current state of research in this field, where high-level randomised controlled trials and large cohort studies are limited. However, this study still provides valuable insights, contributing to the expanding body of literature about this topic. Moreover, most studies had a follow-up duration of 6 months. While this provides an understanding on the short-term effects of HGNS, longer follow-up durations are necessary to fully evaluate its long-term efficacy, safety and sustainability of outcomes. Additionally, our review predominantly included male adults, with fewer female patients represented. This is reflective of the known demographic trends of OSA, where males are consistently reported to have a higher prevalence. Furthermore, up to a certain age, the severity of OSA tends to be higher in males when matched with females for BMI.^42^ However, the underrepresentation of females may limit the generalisability of these findings to both genders. More studies with a balanced gender distribution will help to evaluate HGNS outcomes across different demographic groups.

- Obstructive sleep apnoea (OSA) is a prevalent disorder with significant health implications, including cardiovascular and neurological disorders
- Continuous positive airway pressure (CPAP) is the gold standard treatment for moderate to severe OSA, but poor patient compliance limits its effectiveness
- Alternative therapies have been explored, including hypoglossal nerve stimulation (HGNS), which stimulates the hypoglossal nerve to prevent airway collapse during sleep
- HGNS was initially approved by the United States Food and Drug Administration (FDA) for patients who are intolerant to CPAP and meet specific criteria based on the Stimulation Therapy for Apnea Reduction (STAR) trial: moderate to severe OSA (20 < AHI < 50 events/hour), BMI less than or equal to 32 kg/m^2^ and no soft palate complete concentric collapse (CCC)
- This review finds that HGNS can be effective in patients who fall outside the original STAR trial criteria. Specifically, it shows promising results for patients with higher baseline AHI (> 50 events/hour) and higher BMI (> 32 kg/m^2^), indicating significant reductions in AHI and improvements in daytime sleepiness and quality of life. HGNS could be beneficial for a larger patient population than originally thought
- The study reinforces that HGNS remains less effective for patients with soft palate CCC or significant central apnoea (central apnoea > 25 per cent of total apnoeic events). In these cases, HGNS generally does not lead to substantial improvements in AHI, and outcomes may even worsen for patients with CCC.

## Conclusion

In conclusion, this review suggests the potential of HGNS as an effective treatment for OSA in patients outside the original STAR trial parameters. While these results are promising for patients with AHI and BMI values outside the initial STAR criteria range, caution is warranted in cases involving CCC or a significant central apnoea component as the findings related to these factors remain inconclusive. This underscores the need for further research to evaluate the use of HGNS across a wider range of patient demographics and OSA phenotypes. To optimise outcomes, further refinement of patient selection criteria will be crucial. In this regard, the ADHERE registry holds great potential in fulfilling this purpose.
